# A Framework for Combining rTMS with Behavioral Therapy

**DOI:** 10.3389/fnsys.2016.00082

**Published:** 2016-11-15

**Authors:** K. Zoe Tsagaris, Douglas R. Labar, Dylan J. Edwards

**Affiliations:** ^1^Non-Invasive Brain Stimulation and Human Motor Control Laboratory, Burke Medical Research Institute, White PlainsNY, USA; ^2^Department of Neurology, New York Presbyterian, Weill Cornell Medicine, New YorkNY, USA

**Keywords:** repetitive transcranial magnetic stimulation (rTMS), non-invasive brain stimulation, neurological rehabilitation, behavioral interventions, therapy

## Abstract

Upon its inception, repetitive transcranial magnetic stimulation (rTMS) was delivered at rest, without regard to the potential impact of activity occurring during or around the time of stimulation. rTMS was considered an experimental intervention imposed on the brain; therefore, the myriad features that might suppress or enhance its desired effects had not yet been explored. The field of rTMS has since grown substantially and therapeutic benefits have been reported, albeit with modest and inconsistent improvements. Work in this field accelerated following approval of a psychiatric application (depression), and it is now expanding to other applications and disciplines. In the last decade, experimental enquiry has sought new ways to improve the therapeutic benefits of rTMS, intended to enhance underlying brain reorganization and functional recovery by combining it with behavioral therapy. This concept is appealing, but poorly defined and requires clarity. We provide an overview of how combined rTMS and behavioral therapy has been delineated in the literature, highlighting the diversity of approaches. We outline a framework for study design and reporting such that the effects of this emerging method can be better understood.

## Introduction

The brain is never at rest; the *default-mode network* comprised of coherent and connected brain networks (cingulate cortices, inferior parietal, and medial prefrontal regions), operative at times of behavioral rest, has been examined through the use of blood oxygenation level-dependent (BOLD) signals. Functional MRI testing has shown the default-mode network is active at rest and in the presence of volitional behavior (e.g., repetitive task practice), decreases in activation. This deactivation may allow for associated activity-dependent plasticity in other parts of the motor system. Because of the default-mode network’s activation/deactivation patterns, it appears the motor cortex does not work in isolation during volitional behavior, motor learning, recovery and reorganization (Sanes and Donoghue, 2000; Gusnard and Raichle, 2001; Damoiseaux et al., 2006). Instead, the motor cortex injury-response functional reorganization appears to occur through use-dependent alteration of outputs, which result from behavioral experiences, such as repetitive task practice (Nudo et al., 2001). Interventions promoting recovery should attend to this complex dynamic process, and likely utilize a multi-faceted approach for optimal outcome.

### Background

The initial application of spatially targeted non-invasive brain stimulation (NIBS) as treatment to improve function was not temporally linked to meaningful voluntary brain activity; instead was delivered in isolation of standard therapy practice techniques (Amassian and Maccabee, 2006). Such applications would seem to tap into only a fraction of the complex multi-faceted systems changes involved in learning and memory. Influencing brain areas remote from, but functionally connected to, a primary target, and influencing brain activity at critical time periods relative to practice and therapy timing, may be important additional considerations in this domain.

Hardwired existing pathways (e.g., skilled movement, speech, or executive function) may have lower threshold for activation by transcranial magnetic stimulation (TMS), and therefore be preferentially predisposed to modulation by NIBS. For example, in the motor system, Rothwell et al. (1987) studied a healthy population at rest and found the largest electromyographic (EMG) responses in distal muscles (which have the finest movements associated with them) versus lower EMG responses in proximal muscles. Furthermore, upon simultaneous voluntary contraction, the EMG response latency to brain TMS shortens, becomes larger, and the threshold is lower. Therefore, if voluntary activation is absent or decreased, such as in a diseased brain state, the modulation possible with NIBS may be altered. Conversely, TMS may affect a patient’s ability to produce a voluntary motor activation. Thus the quality and duration of responses seem likely to be influenced by the interactions of interdependent complex systems. The efficacy of stand-alone NIBS is influenced by its stimulation variables, which include: frequency, intensity, coil positioning, stimulation site, and number of sessions delivered (Nollet et al., 2003; Goetz et al., 2016). Therefore, TMS protocols may be adapted to modulate the motor response through excitatory or depressive methods dependent on stimulation parameters, providing opportunity for custom programs based on the population, person, and pathology.

### rTMS and Behavioral Intervention: Current Focus

One contemporary approach is to link NIBS with behavioral techniques, in hopes of producing more robust and durable outcomes. The logic for doing so stems from the idea that effects of repetitive TMS (rTMS) and behavioral therapy will sum or that rTMS will enhance, or consolidate, the effects of therapy. For instance, Mills and Schubert (1995) suggest TMS during sustained voluntary, tonic activity may increase synchronicity of common input fibers and motor neurons. This improved synchrony may promote increased motor cortex plasticity, compared to one intervention alone. Finding the optimal pairing of intensity, duration, frequency, and site, for both rTMS and therapy, would be important for the strongest enhancement, but also to avoid maladaptive plasticity.

Thus one can learn from analyzing the variety of approaches that have been employed previously and assess outcome. In the present paper we aimed to: (a) outline what is meant by *combined therapy* in the context of historical literature [this is examining how traditional therapies (occupational, speech, physical, cognitive-behavioral, or task practice) are combined with rTMS either on or off-line]; (b) provide a snapshot of the literature showing the diverse range of approaches, and outcomes; and (c) propose a framework for how combined therapy should be reported in the literature moving forward.

Most *combined therapy* studies have been on stroke survivors to date and have been intuitively and practically based (**Supplementary Table S1**). For example, time considerations such as equipment practicality, busy clinical setting, staffing, training, and availability, may affect feasibility of temporally combined therapies. Additionally, the participant’s cognitive or physical state during rTMS delivery is rarely reported in the literature but may influence intervention efficacy (e.g., patient engaged in conversation, use of mobile device, listening to music). Despite increasing application of TMS in the field of psychiatry and FDA approval for refractory depression, very little has been done in examining the combination of rTMS with behavioral interventions, such as cognitive behavioral therapy (Micoulaud-Franchi et al., 2013; *note French language*). Instead, most commonly, papers describe using rTMS in isolation for patients with medication-resistant depression (Lam et al., 2008; Slotema et al., 2010). It would be of considerable interest for systematic research on *combined therapy* to expand beyond the motor function domain, and study brain processes such as depression or learning in the future. Hopefully this would lead to a better understanding of broader principles concerning the effects of rTMS as an adjunct to older, traditional, well-accepted therapy approaches.

### Cumulative Effect

Research to determine the cumulative effect and duration of results of NIBS intervention is ongoing. This cumulative effect was examined in a healthy population by Baumer et al. (2003) who found repeated sessions of rTMS (two trains of inhibitory, sub-motor threshold rTMS over the pre-motor cortex) delivered within 24 h, or no greater than 7 days later (consecutive days), induced plastic changes of intrinsic motor cortex excitability. Khedr et al. (2006) and Lomarev et al. (2006) found a cumulative effect of rTMS in individuals with Parkinson’s Disease (eight sessions of 25 Hz rTMS over 4 weeks) which lasted 1 month or more. It appears that multiple sessions of rTMS will lead to a cumulative effect, identified as regions of sustained membrane polarization (Pell et al., 2011). With this effect it may be possible to build on neuromodulatory changes in subsequent sessions to promote recovery or slow disease progression. Similarly, in behavioral therapy, efficacy of treatment may be affected by factors including: duration, intensity, type of intervention, and modality used. These therapeutic variables, specifically intensity and duration of therapy, may carry varying levels of importance dependent on individual functional status, though there is still much debate regarding this (Winstein et al., 2016). Thus there are striking similarities between NIBS and traditional therapy variables, in that frequency, intensity, site or system, and duration of the intervention affect outcome.

## Literature Search

The intent of this search was to identify and summarize published studies combining rTMS with behavioral intervention for clinical benefit. Therefore, we have limited the search to a neurological patient population. All articles were found through the PubMed database and excluded: non-English, case studies, drug studies, transcranial direct current stimulation (tDCS) or other non-invasive stimulation methods, and/or healthy subjects. The acronym *rTMS* was used in combination with the following search terms (relevant/hits): physical therapy (23/267), occupational therapy (8/35), speech therapy (10/54), behavioral intervention (0/95), task training (2/23), motor practice (3/42), motor training (3/50), rehabilitation (8/232), cognition (0/144), cognitive rehabilitation (0/24), cognitive behavioral therapy (2/144), CBT (0/7), cognitive training (0/19). A summary of hits for tDCS in combination with the above search terms is located in **Supplementary Figure S1**, however, will not be further reviewed in this paper.

## Literature Analysis

**FIGURE 1:**
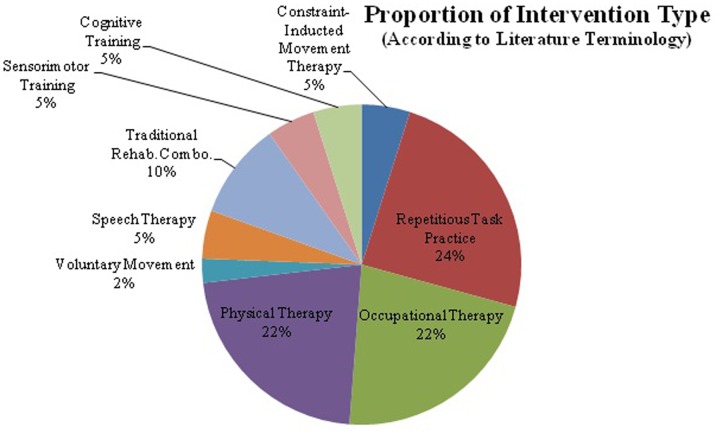
**The majority of research in repetitive transcranial magnetic stimulation (rTMS) and behavioral intervention has been completed with rTMS in conjunction with motor training.** rTMS paired with speech therapy, cognitive training, and sensorimotor training account for only 30%. rTMS in conjunction with cognitive therapies has not been widely studied, despite FDA approval for use in refractory depression. This graph shows the distribution of behavioral interventions used in conjunction with rTMS. Despite there being a fairly wide variety of interventions utilized there are great differences in protocol design and stimulation parameters, which makes comparing protocols or drawing firm conclusions difficult.

The majority of rTMS papers report application of the full stimulation protocol at rest, i.e., not during associated therapy (37/50). A smaller proportion of experimental studies used an interleaved approach of combined therapy, performing the therapeutic task during inter-train intervals (5/50). Less than twenty percent of studies fell into the following categories; unspecified timing (2/50), used high or low frequency dependent on experimental group (3/50), delivered rTMS and therapy simultaneously (2/50) or completed rTMS and therapy on different days (1/50).Within these experimental studies, there was great diversity in the stimulation and therapy variables used for each diagnosis within the neurological population (**Figure 1**). Despite differences, the majority of papers show improvement in clinical outcomes. However, vague and inconsistent reporting of the combined intervention, as well as diverse approaches, impede advancement of understanding toward optimizing intervention and maximizing clinical efficacy. Elements of rTMS application that varied most commonly between studies include; rTMS timing relative to therapy, and either inhibitory (low-frequency), or excitatory (high-frequency) rTMS (**Supplementary Table S1**). Detailed reporting and standardization for both rTMS and therapy characteristics is a strategy for taking into consideration variations between clinicians, locations, resources, and type of intervention.

### Stimulation Targeting

Of studies investigating chronic motor impairment following stroke (24/50), all but one targeted the primary motor cortex, the exception targeted the somatosensory cortex (**Supplementary Table S1**, Study #18). The majority of these studies targeted the unaffected hemisphere (17/24). The remaining studies stimulated the affected hemisphere (5/24; **Supplementary Table S1**, Study #5, 16, 18, 20, 23), or stimulated bilaterally (3/24; **Supplementary Table S1**, Study #6, 9, 13). With regards to pulse frequency in this population, 14/24 stimulated with low, 7/24 with high (**Supplementary Table S1**, Study # 5, 14, 16, 18, 20, 23, 26), and 3/24 alternated between high-frequency stimulation (affected hemisphere) and low-frequency (unaffected hemisphere).

All papers (6/50) addressing motor impairment in a sub-acute stroke population targeted the primary motor cortex. Three studies stimulated the unaffected hemisphere at low-frequency (**Supplementary Table S1**, Study #33, 34, 35), 1 stimulated the affected hemisphere at high-frequency (**Supplementary Table S1**, Study #32), and 1 stimulated both hemispheres (unaffected at low-frequency, affected at high-frequency) dependent on treatment group (**Supplementary Table S1**, Study #31). Cha and Kim (2016) stimulated the cortical representation of the first right dorsal interosseous muscle at a low-frequency in their study examining unilateral neglect and motor control.

Additional studies in the stroke population were completed in acute stroke [>1 month post onset (Emara et al., 2010)] or in sub-acute and chronic patients [3+ months post onset (Chang et al., 2012)]. In the acute population patients were stimulated at high-frequency (affected motor cortex) or low-frequency (unaffected). Chang et al. (2012) stimulated the affected primary motor cortex at high-frequency. Other motor impairment studies were completed in patients with congenital hemiparesis (children), Parkinson’s disease, and hand dystonia (**Supplementary Table S1**, Study # 1, 2, 3, respectively). Gillick et al. (2014) stimulated the unaffected primary motor cortex with priming rTMS (high then low-frequency) for children with congenital hemiparesis. Yang et al. (2013) stimulated the contralateral primary motor cortex at high-frequency to “more affected side” in patients with Parkinson’s disease. Kimberley et al. (2015) stimulated the unaffected pre-motor cortex at low-frequency in patients with hand dystonia. One study (Lim et al., 2010) studied hemispatial neglect and stimulated the left parietal area (P5; affected side for all participants) at low-frequency.

Studies examining speech impairments following stroke (*n* = 11) were completed in sub-acute (<6 months post-onset; 6/11) and chronic populations (>6 months post-onset; 5/11). Within the speech domain, Momosaki et al. (2014) studied oral motor control and stimulated two times daily (low then high-frequency) to the pharyngeal muscles representation. Of studies examining aphasia (*n* = 10), four stimulated Broca’s Area (i.e., pars triangularis); 3/4 delivered a low-frequency (**Supplementary Table S1**, Study #40, 42, 46), while 1/4 stimulated with priming rTMS [high then low-frequency (Khedr et al., 2014)]. Abo et al. (2012) differentially defined the stimulation target based on aphasia-type, as determined by fMRI activation (inferior frontal gyrus in non-fluent aphasia and superior temporal gyrus in fluent aphasia) and stimulated at low-frequency.

Studies targeting cognitive impairment were completed in Alzheimer’s (2/3), and chronic stroke populations (1/3). In the Alzheimer’s studies, high-frequency stimulation was delivered to cortical targets including: Broca’s Area, Wernicke’s Area, right dorsolateral prefrontal cortex, and left parietal somatosensory association cortex (two targets stimulated one day, third target on another) (**Supplementary Table S1**, Study #49, 50). Park and Yoon (2015) targeted the left prefrontal cortex in stroke, the affected hemisphere for all participants, at high-frequency.

Thus no clearly superior targeting strategy has emerged. Stimulation of a primary lesion area, stimulation of other areas with significant network connections to a primary lesion area, and stimulation of a focal area in the presence of a widespread brain disease process, all seem effective.

### Outcomes with Respect to Timing

The relative timing of combined therapy has not been well explored, and is one of the most poorly reported variables in rTMS studies; however, none of the papers examined in this review reported a negative response, regardless of timing. In papers examining rTMS prior to behavioral intervention, 86.5% (32/37) cited a positive response, while 5/37 (**Supplementary Table S1**, Study #3, 20, 28, 35, 42) report a neutral response. The second most frequent approach (5/50) involved alternating protocols (behavioral intervention during the inter-train interval), all of which indicate a positive result in outcome (**Supplementary Table S1**, Study #5, 23, 30, 31, 32). Out of the remaining 20% of identified studies, 7/8 reported a positive response (**Supplementary Table S1**, Study #1, 10, 16, 26, 46, 49, 50), while 1/10 reported a neutral response (**Supplementary Table S1**, Study #48). Thus, as in the case of stimulation targeting, discussed above, no clearly superior strategy for temporal pairing of rTMS and behavioral therapy has emerged. However, firm conclusions cannot be drawn due to small sample sizes and need for additional randomized, controlled clinical trials.

**FIGURE 2:**
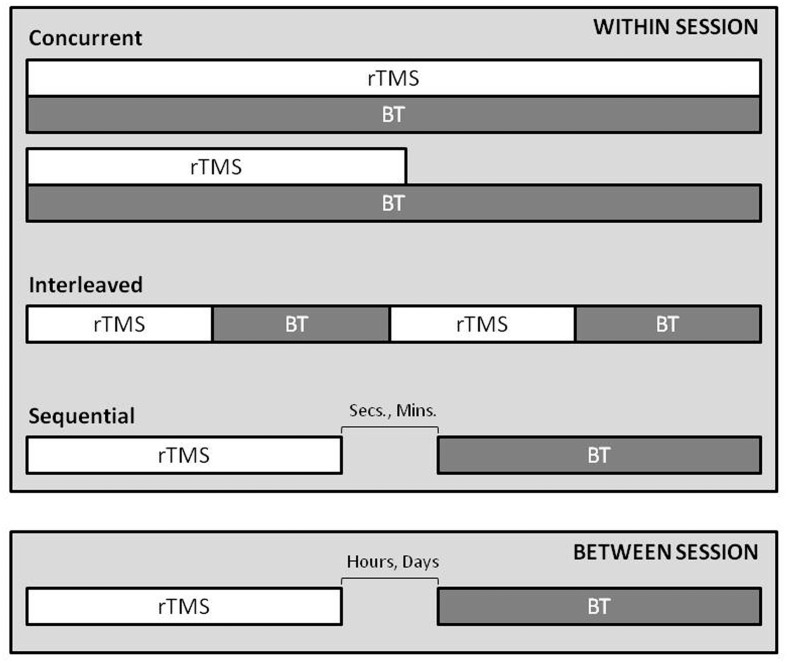
**A conceptual guide for timing of rTMS and behavioral therapy.** The within session and between session temporal relationship, influences *during* and *after*-e*ffect* interactions, depending on the time-course of combination. The relationship can be defined as; 1. *concurrent*, rTMS is applied at the same time as the behavior is expressed (reporting should include if one intervention outlasts the other), 2. *interleaved*, rTMS trains are alternating with behavioral expression/repetition, 3. *sequential*, one intervention follows the other. Note: Separation of the two techniques by hours or days may not harness the interactions of short term after effects of each.

For future research and reporting, we propose the temporal relationship of rTMS application with behavioral intervention be defined as: *concurrent* (rTMS being applied at the same time as the behavior is expressed); *sequential* (one intervention follows the other), *interleaved* (rTMS trains are alternating with behavioral expression/repetition) (**Figure 2**).

One could consider an ongoing pharmacological intervention paired with rTMS a concurrent application of combined therapy, and studies are underway of this nature. Sequential stimulation can occur at numerous time points prior to or following therapy (e.g., volitional activity), ranging from seconds to days. For instance, pulses can be delivered in an event-triggered manner, perhaps with an electroencephalogram (EEG) defined trigger; where important EEG changes known within the neural systems are presently under investigation. Therapeutic rTMS could plausibly be delivered seconds before a task through a clinician prompted visual or verbal cue to promote cognitive effort or movement. Indeed, a single case with positive outcome was reported in the literature for depression where rTMS was combined with a form of cognitive behavioral therapy; here, the cognitive effort was performed in between trains of rTMS – so in this case, combined, but alternating (Vedeniapin et al., 2010). Behavioral intervention can be initiated within minutes following stimulation through use of a defined time window to ensure consistency [i.e., therapy starts 5–10 min after rTMS completion, as in clinical trial NCT02089464 (Nexstim Ltd, 2015)]. Stimulation can be delivered within hours or days of therapeutic intervention (i.e., rTMS in morning, therapy in the afternoon or next day). Optimal timing may vary depending on diagnosis and behavioral intervention type, and further research is needed in this area, as 74% of papers delivered rTMS before behavioral intervention (37/50).

While the physiologic effects of rTMS and behavioral therapy are likely different; a key commonality in both is that after-effects have been linked to adaptive behavioral response, and attributed to lasting modification of synaptic strength in cortical networks subserving the behavior (Butefisch et al., 1995; Silasi and Murphy, 2014), which can be local (e.g., primary motor cortex), or distant through functionally connected networks. The physiological interaction of brain stimulation and discrete voluntary behaviors has been well reported experimentally in healthy subjects (Iyer et al., 2003; Daskalakis et al., 2006; Buch et al., 2011), with striking interactive effects, including augmentation, cancelation, and reversal of effect, depending on the circumstances of the interaction. In the context of the present review, the data suggest a more uniform positive response to intervention, which may be troubling. The authors suggest a framework for reporting research methods in studies examining paired rTMS and behavioral interventions (**Supplementary Table S2**).

### rTMS and Behavioral Therapy in Other Functional Domains and Adverse Events

Currently there is a lack of research on combined applications studying functional domains outside of motor and speech. In the future, rTMS may be a promising approach for increasing the efficacy of currently accepted, traditional behavioral interventions. There were no serious adverse events reported in our reviewed studies, and only mild headaches were reported in a few papers.

## Conclusion

Combined therapy of rTMS paired with behavioral intervention has gained traction in the scientific field. Historically, rTMS was delivered in isolation, yet with positive results. Researchers are now examining how rTMS may be used as an adjuvant to more traditional therapies (e.g., physical, occupational, speech, or cognitive therapies) to maximize the benefit of both interventions. To date, the majority of research has been completed in the motor-domain of a stroke population and has examined delivering rTMS prior to the behavioral intervention. Of the studies reviewed, combined therapy appears to be safe, since only minor adverse effects (primarily mild headache) were reported in a few papers. Further research is needed to examine the optimal pairing of rTMS and behavioral intervention. Focused attention to all rTMS parameters and timing of stimulation is essential to allow for study replication and data interpretation. Similarly, therapy-related specifications must be clearly reported and standardized. In order to determine optimal combined therapy, larger sample sizes and randomized, controlled clinical trials are needed to account for variability among individuals and conditions.

## Author Contributions

KT contributed the acquisition of data and drafting of manuscript. DL and DE contributed study conception and design, as well as critical revision.

## Conflict of Interest Statement

The authors declare that the research was conducted in the absence of any commercial or financial relationships that could be construed as a potential conflict of interest.
