# Supplementary material for: A Framework for Combining rTMS with Behavioral Therapy
Source: Front Syst Neurosci. 2016 Nov 15;10:82. doi: 10.3389/fnsys.2016.00082 (PMC5108775; doi:10.3389/fnsys.2016.00082)
Supplement: Supplementary file 1 [file Data_Sheet_1.docx]

| **Authors**  *Table S1* | **Timing & Total Duration** | **Frequency of TMS** | **TMS Specs** | **Stimulation Site** | **Therapy Specs** | **Impact** |
| --- | --- | --- | --- | --- | --- | --- |
| **1.** Gillick, Krach, Feyma, Rich, Moberg,Thomas, et al. (2014)  Children with congenital hemiparesis,  Motor | rTMS then therapy next day  N=19 | High then Low | 5 treatments of rTMS  Priming rTMS  (10 min of 6 Hz rTMS at 90% rMT. Total of 600 low frequency pulses) then  1Hz rTMS | Contralesional primary motor cortex | 5 treatments of CIMT (shaping, repetitive activities for function, ROM, strengthening of UE) in total. 2 hours with a trained therapist  Constraint cast applied on tx day 2 and removed on tx day 10 (cast worn for total of 13 days incl. weekends) | (+) Combining 6 Hz priming and LF rTMS with CIMT appears to provide an added benefit above CIMT alone; significant functional outcomes and well tolerated- suggests synergistic effect of rTMS & CIMT having the potential to improve efficacy of neurorehabilitation applications in this population. |
| **2.** Yang, et al. (2013)  Parkinsons, Motor | rTMS then therapy  N=22 | High | 12 sessions over 4 weeks (3x/week)  5 Hz (6 min), 1200 pulses, 100% of rMT  Train duration: 10 s with 5 sec inter-train interval | Tibialis anterior muscle of motor cortex, Contralateral to more affected side. | Motorized treadmill speed 80% of overground comfortable walking speed, increased by increment of 0.2 km/h per 5 min as tolerated.  Total 30 min | (+) Combination of rTMS and treadmill training enhances the effect of treadmill training on modulation of corticomotor inhibition and improvement of walking performance in those with PD. |
| **3.** Kimberley, Schmidt, Chen, Dykstra, Buetefisch  (2015)  Hand dystonia, Motor | rTMS then therapy  N=9 | Low | 5 intervention sessions  Participants completed both groups with 1 month wash out period  1200 pulses (20 min), 1 Hz 80% of rMT  Group 1: rTMS + non-specific stretching/massage (control therapy)  Group 2: rTMS + sensorimotor | Contralesional Pre-motor cortex (first dorsal interosseous muscle) | Sensorimotor training program (30 min)  Sensory discrimination training | (N) There was no superior benefit or neurophysiologic change to the sensorimotor retraining intervention with rTMS compared to control with rTMS. |
| **4.** Lim, Kang, Paik (2010)  Acute stroke, unilateral right hemispheric stroke, hemispatial neglect | rTMS then therapy  N= 7 | Low | 10 daily sessions (5x per week for 2 weeks)  1 Hz at 90% of rMT for 15 min (900 pulses) | Left parietal area (P5) | 30 min behavioral therapy- standardized neglect therapy protocol  Emphasis on top-down approach including visual scanning and feedback provision. | (+) Inhibitory low-frequency rTMS is a safe treatment modality and rTMS applied over the healthy left parietal area before behavioral therapy improved line bisection test results compared with behavioral therapy alone. |
| **5.** Koganemaru et al. (2015)  Chronic stroke,  Motor | Alternating  (1 train TMS, 1 cycle exercise)  E-Stim during the movement  N=11 | High | 15 cycles  15 Hz for 8 sec, interval of 52 sec, 100% of the active motor threshold  Addt’l stimulation (neuromuscularm, EEx) EDC muscle belly and distal tendon.  40 Hz train of 250 µs^2^ constant current pulses, lasting for 500 ms (20 pulses) | Ipsilesional M1 | 15 cycles of exercises for wrist/finger extensors  Each cycle: 50 s of exercise followed by a train of TMS  Exercise: 50 repeats of 1 Hz rhythmic voluntary extension of the wrist and MCP joints of the 5 digits  Participants asked to make 2/3 AROM extension at the *same time* as e-stim | (+) Combining motor training with rTMS can facilitiate use-dependent plasticity and achieve functional recovery of motor impairments that cannot be attained by either intervention alone. |
| **6.** Takeuchi, Tada, Matsuo, Ikoma (2009)  Chronic subcortical stroke,  Motor | rTMS then therapy  N=30 | Low over unaffected  High over affected | Bilateral rTMS:  1 Hz rTMS over unaffected hemisphere (50 sec), alternating with  10 Hz & 5 sec train duration over the affected hemisphere with an interval of 5 sec. (repeat 20 times)  Unaffected: rTMS applied over unaffected hemisphere and sham was applied over affected (same frequency/intensity as bilateral)  Affected rTMS group: rTMS applied over affected hem & sham over unaffected hem | M1 | Pinching task: 15 min- metronome paced pinch of index finger & thumb (affected hand), as fast as possible | (+) The combination of 1 Hz rTMS over the unaffected hemisphere and 10 Hz rTMS over the affected hemisphere could lead to an improvement in the motor function of the paretic hand of patients with chronic stroke. |
| **7.** Barros Galvao et al. (2014)  Chronic stroke,  Motor | rTMS then therapy  N= 20 | Low | 1 session per day (x10)  1 Hz on the unaffected hemisphere, 1500 pulses, intensity of 90% of MT. | Contralesional M1 | 30 min, 3 days/week  Activities to improve strength, flexibility, transfers, posture, balance, coordination and provide sensory stimulation (mainly UE movements) incl. movements of shoulder, wrist, hand, and fingers. | (+) Inhibitory rTMS over un.affected hemisphere in association with PT reduces the spastic hypertonia in the UE, and the effect was maintained for at least 4 weeks after the rTMS sessions. |
| **8.** Etoh, et al. (2013)  Chronic stroke, Motor | rTMS then therapy  N=18 | Low | 1x daily for 5 days/week  Group #1: Motor (2 wks)-before-sham (2 wks)  Group #2: Sham (2 wks)-before-motor (2 wks)  Motor: 1 Hz for 4 min (240 pulses, 90% of rMT) | Contralesional motor cortex over abductor pollicus brevis (APB) | Voluntary training without assistance of a PT/OT for 1-2 hrs  Repetitive facilitation exercises | (+) Multiple sessions of 1 Hz rTMS facilitated the effects of RFEs in improving motor function of the affected upper limb but did not change spasticity in chronic stroke patients. |
| **9.** Kakuda, et al. (2011)  *Neurorehabilitation*  Chronic stroke, Motor | rTMS then therapy  N=11 | High then Low | 15 day protocol:  10 min intermittent 6 Hz rTMS applied in 5-sec trains, 25 sec intervals between trains (total 600 pulses).  Immediately after: 1 Hz applied for 20 min, 90% of MT (total 1200 pulses) | First dorsal interoseous (FDI) in unaffected UE | 60 minute 1 on 1 training, 60 minute self training (no supervision).  Incl. shaping techniques, repetitive task practice techniques  Intervention varied based on level of function/interest. | (+) rTMS with priming stim has more potent long-standing suppressive effect than LF rTMS alone.  Fugl-Meyer scores increased significantly  WMFT time shortened significantly  Functional ability score increased significantly |
| **10.** Kakuda, Abo, Kobayashi, Momosaki, Yokoi, Fakuda, et al. (2011) *Brain Injury*  Chronic stroke, Motor | Order not specified  N=39 | Low | 15 day protocol, 22 treatment sessions (1-2 sessions daily)  1 Hz rTMS, 1200 pulses (20 min) 90% of MT of FDI muscle | Contralesional hemisphere at site that elicited largest MEP for first dorsal interosseous (FDU) | 120 minute OT:  60 min 1 on 1 training (Incl. shaping techniques, repetitive task practice techniques)  60 min self training  Intervention varied based on level of function/interest. | (+) low frequency rTMS/OT protocol significantly decreased the MAS scores for both finger and wrist flexors at discharge and 4 weeks after discharge. The protocol also increased Fugl-Meyer Assessment score and shortened the WMFT performance time. |
| **11.** Kakuda, et al. (2010)  *Int’l J Rehabil Res*  Chronic stroke, Motor | rTMS then therapy  N=15 | Low | 15 day protocol:  1 Hz, 90% of MT, 1200 pulses. | Contralesional hemisphere- first dorsal interosseous muscle of unaffected UE | 60 min 1 on 1 training  60 min self training  Shaping techniques, stretching, facilitation techniques, manual dexterity techniques, repetitive task techniques, performed with restraint of unaffected UE using cotton hemi-sling | (+) rTMS combined with intensive OT over 15 day period was safe and resulted in improvement of motor function and attenuation of spasticity of the affected UE in post stroke patients. |
| **12.** Kakuda, et al (2010)  *Disability & Rehab*  Chronic stroke, Motor | rTMS then therapy  N=5 | Low | 1 Hz rTMS, 90% of MT,  1200 pulses (20 min) | Contralesional hemisphere over primary motor area | After rTMS  6 consecutive days, 10 sessions:  Day 1 & 6- 1 session  Day 2-5- 2 sessions.  1 hr one on one training (w/ restraint of unaffected UE)- shaping techniques, repetitive task practice technique.  1 hr self-training | (+) motor improvement in the affected UE |
| **13.** Yamada, et al. (2013)  Chronic stroke, Motor | rTMS then therapy  N=8 | Low then High | 1 train, bihemispheric rTMS, 50 sec period of 1 Hz rTMS to nonlesional hemisphere, 5 sec period of 10 Hz rTMS applied to lesional hemisphere. Interval of 5 sec set between the two rTMS simulations  (100 stimulations in total in each train of 60s).  40 trains of bihemispheric  1 daily session rTMS= 40 min, 90% MT | FDI muscle of both UE | 120 min one to one training sessions (shaping techniques & repetitive task practice techniques).  120 min of self training | (+) Mean FMA score increased significantly , reduction in performance time of WMFT |
| **14.** Kakuda, Abo, Watanabe et al. (2013)  Chronic stroke, Motor | rTMS then therapy  N=19 | High | 20 sessions over a 13-day period  10 Hz rTMS applied in 10 sec trains (100 pulses per train) with 50 sec intervals between trains. (2000 pulses/session, 20 min), 90% of rMT | Tibialis anterior of unaffected lower limb | 60 min of task-related and specific mobility training (1 on one).  Warm up, stretching (5-10 min), sit to stand (5-10 min) and treadmill training (25-35 min) | (+) high frequency rTMS over bilateral leg motor areas and mobility training is safe/feasible for post-stroke hemiparetic patients with gait disturbance. significant improvements in mobility |
| **15.** Kakuda, et al. (2012)  Chronic stroke, Motor | rTMS then therapy  N=204 | Low | 22 treatment sessions (15 days)  2 sessions per day except for the days of admission/discharge and Sundays  1 Hz rTMS, 1200 pulses (20 min), 90% of rMT of FDI muscle | Contralesional hemisphere over M1 (FDI muscle of unaffected UE) | Started within 10 minutes of rTMS completion.  120 minutes of occupational therapy  60 min 1:1 training  60 min self-exercise  (shaping & repetitive task practice techniques)  +positive verbal guidance | (+) significant improvement of motor function of the affected UE in post-stroke patients  Response to tx not influenced by age or time since stroke onset. |
| **16.** Kwon, Kim, Chang, Bang, Shin (2014)  Chronic stroke, Motor | rTMS then Therapy (mixed)  rTMS then therapy (not mixed)  N= 14 | High | 2 sessions, at least 48 hrs in between  “Interleaved combination method” (fifty 10 Hz rTMS trains at 90% rMT applied for 5 seconds over motor area. Train repeated 20 times with a 55 second inter-train interval, a total of 1000 pulses delivered in 20 min session)  “preconditioned combination method” (100 trains of 10 Hz rTMS at 90% rMT. Train repeated 10 times with a 50 second inter-train interval. Total of 1000 pulses delivered over 10 minutes) | Motor cortex area of paretic hand | Motor training  “Interleaved combination method”  After each inter-train interval, patients practiced a block of sequential finger motor tasks for 20 seconds.  400 seconds total were devoted to sequential motor task during each session.  “Preconditioned combination method”  After rTMS, practice for 10 minutes, 40 second blocks of sequential finger m otor tasks followed by a 20 sec. rest period. 400 seconds devoted to task practice | (+) In combining rTMS and motor training, preconditioning with rTMS followed by motor training is more effective than interleaving the rTMS & motor training to facilitate the motor performance of stroke patients. |
| **17.** Wang, Tseng, Liao, Wang, Lai, Yang (2012)  Chronic stroke, Motor | rTMS then therapy  N=24 | Low | 10 sessions over 2 weeks  90% of rMT, train of 600 pulses (1 Hz) for 10 min.  OR SHAM | Leg area of motor cortex, unaffected hemisphere | 6 workstations (5 min each) for a total of 30 min.  Included: standing and reaching, sit to stand, stepping up, heel lift exercise, up and go exercise, ambulation training | (+) rTMS enhances the effect of task-oriented training in those with chronic stroke, especially by increasing gait spatial symmetry and coritcomotor excitability symmetry |
| **18.** Brodie, Meehan, Borich, Boyd (2014)  Chronic stroke, Motor | rTMS then therapy  N=15 | High | 7 sessions total separated by no more than 3 days.  24 trains, 5 Hz rTMS, 90% RMT for 10 seconds, with 5 seconds rest in between (1200 pulses in total) | IL-S1 | 5 sessions  Serial Tracking Task- using hemiparetic hand to control mouse and answer prompts on screen  Each block: 9 alternating reps of 8 element sequences (5 random- assessed changes in non-specific motor control, 4 repeated- effects on implicit motor sequence learning)  *Duration of each block dependent on individual performance- avg 4 min. | (+) 5 Hz rTMS over IL-S1 paired with skilled motor practice enhanced motor performance and learning of a novel skilled motor task in individuals with chronic stroke. Benefits also associated with significant improvements in somatosensation |
| **19.** Wang, Tseng, Liao, Wang, Lai, Yang (2012)  Chronic stroke, Motor | rTMS then therapy  N= 28 | Low | 10 sessions (consecutive weekdays)  90% rMT, 600 pulses (1 Hz) for 10 min.  Or sham rTMS + therapy | Leg area of motor cortex, unaffected hemisphere | 30 min task oriented training session delivered by physical therapist (6 workstations, each attended for 5 min.)  Each individual participated in 1:1 therapy- difficulty of task or repetitions increased with improvements made | (+) rTMS combined with task-oriented training is beneficial for motor recovery and brain reorganization in chronic stroke patients. |
| **20.** Malcolm, Triggs, Light, Gonzalez Rothi, Wu, Reid, Nadeau  (2007)  Chronic stroke, motor | rTMS then therapy  N=19 | High | 10 sessions (consecutive weekdays, 2 weeks)  2000 pulses (50 trains of 40 pulses), 20 Hz, 90% of rMT. Interrtrain interval of 28 secs.  Or Sham | Hand area of motor cortex, affected hemisphere | Onsite training: Constraint induced therapy (mitt on unaffected limb) and engaged in variety of functional tasks directed at the affected upper limb.  Home practice: Following session, participant completed 5 hours  Mitt worn for 90% of waking hours. | (N) Although this study provided further evidence that even relatively brief sessions of CIT can have a substantial effect, it provided no support for adjuvant use of rTMS |
| **21.** Takeuchi, Tada, Toshima, Chuma, Matsuo, Ikoma (2008)  Chronic stroke, subcortical type only, Motor | rTMS then therapy  N=20 | Low | 1 session  rTMS delivered at 1 Hz, 90% of rMT for 25 minutes (1500 pulses) or sham rTMS. | Motor cortex of the unaffected hemisphere | Pinching task (metrobnome-paced pinch task of their index finger thumb of the affected hand as fast as possible) for 15 minutes | (+) rTMS induced an increase in the excitability of the affected motor cortex and an improvement in acceleration of the affected hand. Moreover, the effect of motor training on pinch force was enhanced by rTMS. These improvement in the motor function lasted 1 week after RTMS and motor training. |
| **22.** Kakuda, Abo, Kobayashi, Takagishi, Momosaki, Yokoi, Fukuda, Ito, Tominaga (2011)  Chronic stroke, Motor | rTMS then therapy  N=52 | Low | 22 treatment sessions over 15 days.  1200 pulses, 1 Hz, 90% or MT | First dorsal interosseous muscle (FDI) of unaffected limb | 120 min of OT.  60 minutes of self training, 60 minutes of 1 on 1 training  Consisting of: shaping techniques, repetitive task practice, facilitation techniques, manual dexterity | (+)The extent of motor improvement by the intervention seemed to be influenced by the severity of upper limb hemiparesis at study entry. |
| **23.** Kim, You, Ko, Park, Lee, Jang, Yoo, Hallett (2006)  Chronic Stroke, Motor | Intermittent rTMS and therapy  N=15 | High | 2 sessions with 1 week interval  ( 1 session real, 1 session sham in pseudorandomized order)  20 pulses at 10 Hz, 80% of Rmt (total duration= 2 sec). Train was repeated 8 times, total of 160 pulses delivered. 8 minute session with a 58 second intertrain interval.  Or Sham | M1 of affected hemisphere | Block of sequential finger motor tasks for 40 seconds during the intertrain intervals.  Motor practice task block was repeated 8 times. | (+) High frequency rTMS resulted in a significantly larger increase in the MEP amplitude than the sham rTMS, and the plastic change was positively associated with an enhanced motor performance accuracy. |
| **24.** Barros Galvao, Costa dos Santos, Borba dos Santos, Cabral, Monte-Silva (2014)  Chronic stroke, Motor | rTMS then therapy  N=20 | Low | 1 session per day for 10 sessions.  1 Hz, 1500 pulses at 90% of MT.  OR SHAM | M1, Unaffected hemisphere | Approx. 30 minutes, 3 days per week  Focused on: improving strength, flexibility, transfers, posture, balance, coordination. Also included sensory stimulation, upper limb movements. | (+) rTMS associated with PT can be beneficial in reducing poststroke spasticity. |
| **25.** Vongvaivanichakul, Tretriluxana, et al (2014) *J Med Assoc Thai*  Chronic stroke, Motor | rTMS then therapy  N=14 | Low | 6 sessions  1 Hz, 90% of rMT for 20 min (1200 pulses)  OR SHAM | Abductor pollicus brevis muscle at M1 of the non-lesioned hemisphere | Reach to grasp training: grasp and release a can at 15 cm anterioraly from their hand, frequency 12 times per minute  Training 5 minutes, resting 2 minutes per 1 session | (+) LF-rTMS and RTG training enhanced the training effect as evidenced by faster movement in the desterity tasks of the paretic hand than RTG training alone. |
| **26.** Kwon, Kim, Chang, Bang, Shin (2014)  Chronic stroke, motor | Interleaved OR  rTMS then therapy  N=14 | High | 2 experimental sessions (at least 48 hrs in between)  Interleaved combination method:  50 10-Hz rTMS trains at 90% rMT. Train repeated 20 times with 55 second inter-train interval  Preconditioned combination method:  100 trains of 10 Hz rTMS at 90% of rMT. Train repeated 10 times with a 50 second inter-train interval (total of 1000 pulses delivered over 10 min) | Contralesional motor cortex | Interleaved combination method:  Practiced a block of sequential finger motor tasks for 20 seconds immediately after each rTMS train (total of 400 seconds)  Preconditioned combination method:  After rTMS, practice for 10 minutes, 40 secs blocks of sequential finger motor tasks followed by 20 secs rest period (total of 400 secs) | (+) In combining rTMS and motor training, preconditioning with rTMS followed by motor training is more effective than interleaving the rTMS and motor learning to facilitate the motor performance of stroke patients. |
| **27.** Avenanti, Coccia, Ladavas, Provinciali, Ceravolo (2012)  Chronic stroke, Motor | rTMS then therapy  N=30 | Low | 10 days  Single train of 1500 pulses, 90% rMT (25 minutes)  OR SHAM | First dorsal interosseous (FDI) in the intact motor cortex | 45 minutes of Physical therapy focused on: Daily routine tasks, finger force, task-oriented exercises, key grip. | (+) Priming PT with inhibitory rTMS is optimal to boost use dependent plasticity and rebalance motor excitability and suggest that time-locked rTMS is a valid and promising approach for chronic stroke patients with mild motor impairment. |
| **28.** Rose, Patten, McGuirk, Lu, Triggs (2014)  Chronic stroke, Motor | rTMS then therapy  N= 22 | Low | 4 times per week for 4 weeks (16 sessions)  Total of 1200 pulses delivered as a single 1 Hz train, 100% of rMT  OR SHAM | Contralesional M1 corresponding to extensor carpi radialist (ECR) | 1 hour of task practice  Included: reaching, grasping, manipulation of objects performed with paretic UE only. | (N) rTMS did not augment changes in UE motor ability in this population of individuals with chronic stroke. The chronicity of our participant cohort and their degree of UE motor impairment may have contributed to inability to produce marked effects using rTMS. |
| **29**. Emara, Moustafa, ElNahas, ElGanzoury, Abdo, Mohamed, et al. (2010)  Stroke (>1 month post onset), Motor | rTMS then therapy  N= 60 | Low & High | 10 sessions over 10 days  Group 1: Sham rTMS  Group 2: Ipsilesional 5 Hz rTMS, continuous 2.5 min train at 80-90% of MT, 750 pulses per session  Group 3: Contralesional 1 Hz rTMS, continuous 2.5 min train at 110-120% of MT, 150 pulses per session | Ipsilesional hemisphere hotspot (thumb abductor),  Contralesional hemisphere hotspot (thumb abductor) | Standard physical therapy. Custom rehab. Protocol for fine hand movement was devised. Completed under supervision of specialized therapist. | (+) rTMS has beneficial effects on motor recovery that can be translated to clinically meaningful improvement in disability in patients with post-stroke hemiparesis, with a well-sustained effect. |
| **30.** Chang, Kim, Yoo, Goo, Park, Kim, Pascual-Leone (2012)  Subacute/  Chronic Stroke (3+ months), Motor | Alternating  TMS then therapy during intertrain intervals  N=21 | High | 10 daily training sessions, 5x/week for 2 consecutive weeks  50 pulses of 10 Hz rTMS applied, 55 sec intertrain intervals (1000 stimuli total during 20 min of training) | Affected M1 | 50 second task blocks, 10x, with a pause of 5 seconds after each task. sequential finger motor learning tasks | (+) significant improvement in movement accuracy after completion of rTMS & motor training with interaction effect between time and intervention |
| **31.** Kim, Choi, Jung, Lee, Lee, Lim (2014)  Subacute Stroke, Motor | Motor training between trains of rTMS, then OT after rTMS finished  N= 40 | Low & High | 10 treatment sessions (2 weeks)  2 treatment groups  Low Frequency: 1 Hz, 120% of rMT for 150 seconds, then rested for 30 seconds. Repeated 10 times for total of 1500 pulses.  High Frequency: 20 Hz, 90% of rMT for 5 seconds then rested for 50 seconds. Repeated 20 times for a total of 2000 pulses. | Low frequency: M1 of contralesional hemisphere  High frequency: M1 of the ipsilesional cerebral hemisphere) | During rest period of rTMS: exercise training (AROM, holding/moving/releasing cups) completed.  Occupational therapy right after rTMS for 60 minutes (incl. ROM training, muscle exercise, fitness training, gait training, and ADL training) | (N) There was no significant difference in motor function of the affected upper extremity between 1 Hz and 20 Hz rTMS during the subacute period of ischemic stroke. |
| **32.** Chang, Kim, Bang, Kim, Park, Lee (2010)  Subacute stroke, Motor | Interleaved  N=28 | High | 10 sessions over 2 week period  50 trains of 10 Hz for 5 seconds at 90% rMT. Total of 1000 pulses delivered with a 55 sec. inter-train interval consisting of 50 sec of motor training and 5 sec of rest.  OR SHAM | Primary motor cortex of affected hemisphere | Active and assistive range of motion of affected extremity, grasp, move, and release of cups and cubes.  In addition, participants received 3 hrs of skilled therapy per day. | (+) Positive long term effects on motor recovery could be achieved after 10 daily sessions of high frequency rTMS in conjunction with motor practice during the subacute period of stroke. |
| **33.** Conforto, Anjos, Saposnik, Mello, Nagaya, Santos, Ferreiro, Melo, Reis, Scaff, Cohen (2012)  Subacute stroke, motor | rTMS then therapy  N=30 | Low | 5 days per week, for 2 weeks (10 sessions)  1 Hz rTMS administered at 90% of rMT for APB for 25 min (1,500 pulses)  OR SHAM | Nonparetic hand abductor pollicis brevis muscle | 60 min. customary outpatient rehabilitation treatment | (+) Low frequency rTMS to the contralesional motor cortex early after stroke is feasible, safe and potentially effective to improve function of the paretic hand, in patients with mild to severe hemiparesis |
| **34.** Zheng, Liao, Xia (2015)  Subacute stroke, Motor | rTMS then therapy  N=112 | Low | 6 times per week (24 sessions)  1800 pulses, 1 Hz rTMS, 90% of rMT (30 min)  OR SHAM | Contralesional M1 (first dorsal interosseous, FDI) | 1 hour of PT, 30 min OT, 30 min VR training per day  Virtual reality graded training program providing goal oriented reaching and/or grasping exercises (started within 10 min. following rTMS) | (+) Combined use of LF rTMS with VR training could effectively improve the upper limb function, the living activity, and the quality of life in patients with hemiplegia following subacute stroke, which may provide a better rehab treatment for subacute stroke. |
| **35.** Seniow, Bilik, Lesniak, Waldowski, Iwanski, Czlonkowska (2012)  Subacute stroke, Motor | rTMS then therapy  N=40 | Low | 15 once-daily sessions, 5 days per week for 3 weeks.  30 min. rTMS: 90% of rMT, 1 Hz, total of 1800 pulses  OR SHAM | Hand area of M1 in the unaffected hemisphere (first dorsal interosseous muscle) | 45 min. Physical therapy focused on neurodevelopmental treatment/Bobath concept. Included active and active assisted exercises of affected hand, gait training, practice of activities of daily living | (N) Findings did not suggest that rTMS suppression of the contralesional motor cortex augments the effect of early neurorehab. For upper limb hemiparesis. |
| **36.** Cha, Kim (2016)  Subacute stroke, unilateral neglect/motor | rTMS then therapy  N=30 | Low | 5 sessions per week for 4 weeks (20 sessions)  1 Hz for 5 min at 90% of rMT (performed 4 times, total of 1200 pulses)  OR SHAM | First dorsal interosseous muscle on the right side | 30 min. of conventional | (+) rTMS might be effective in improvement in reduction of the unilateral neglect and motor function |
| **37.** Abo, Kakuda, Watanabe, Morooka, Kawakami, Senoo (2012)  Chronic left hemiparetic stroke & aphasia | rTMS then therapy  N=24 | Low | 11 day hospitalization, 10 treatment sessions (single session per day).  1 Hz, 90% of rMT of left thenar muscles, 2400 pulses lasting 40 minutes. | Right motor cortex  fMRI used to determine stim. Locations (most activated hemisphere= compensatory hem. for impaired language function—inhibitory)  Applied to IFG of frontal lobe in nonfluent aphasia and to the STG of temporal lobe for fluent aphasia. | 60 minute training session, 1:1 by speech therapist. Goal to improve expressive modality including word production (spontaneous speech), repetition, naming, and writing. Individualized program based on severity of aphasia. | (+) Nonfluent aphasic patients showed significant improvement of auditory comprehension, reading comprehension, and repetition. Fluent aphasic patients showed significant improvement in spontaneous speech only. |
| **38.** Weiduschat et al. (2011)  Subacute stroke, Speech | rTMS then therapy  N=14 | Low | Sessions conducted 5x/week for a 2 week period, yielding 8-10 sessions per subject (mean 9.2  1 Hz rTMS (20 min), 90% of motor threshold. | Right triangular part of the inferior frontal gyrus | 45 min of Model-oriented aphasia therapy focused on individual linguistic problems. Tasks selected assumed to activate left hemipsheric language areas. | (+) inhibitory rTMS of right hemispheric Broca homolog together with speech therapy prevents establishing right hemispheric lateralization and this normalization of the activation pattern may be accompanied by better clinical improvement. |
| **39.** Khedr, El-Fetoh, Ali, El-Hammady, Khalifa, Atta, et al (2014)  Subacute stroke, Speech (non-fluent aphasia) | rTMS then therapy  N= 30 | Low then high | 10 sessions (5x/week)  Sequential stimulation of either hemisphere  One continuous 1 Hz train at 110% of rMT over unaffected right Broca’s area100 total pulses)  THEN  10 trains 20 Hz stimulation, 80% rMT, each lasting 5 seconds with inter-train interval of 30 seconds over left Broca’s area of affected hem. | Broca’s area of affected hemisphere(5 trains over pars opercularis, 5 trains over pars triangularis) , Broca’s area of unaffected hemisphere (500 pulses over pars opercularis, 500 pulses over pars triangularis) | 45 minutes Subtests of Boston Diagnostic Aphasia Examination used for training: naming, repetition, auditory comprehension  Training delivered by a speech and language pathologist | (+) significantly greater improvement in the Hemispheric Stroke Scale (HSS) language score and SADQ-H (stroke aphasic depression questionnaire- hospital version) after real rTMS instead of sham. |
| **40.** Waldowski, Seniow, Lesniak, Iwanski, Czlonkowska (2012)  Subacute stroke, Speech (aphasia) | rTMS then therapy  N= 26 | Low | Mon- Friday for 3 weeks (15 sessions)  1 Hz, 90% of rMT of First dorsal interosseous muscle of the unaffected hand  Applied for 30 min (15 min over PTr, 15 min over POp)  Or Sham rTMS | Right frontal language homologue  Pars triangularis (PTr), pars opercularis (POp) | 45 minutes of speech therapy  Focused on expression and comprehension of spoken language rather than written language. | (+) Inhibitory rTMS of the unaffected right inferior frontal gyrus area in combination with speech & language therapy cannot be assumed as an effective method for all poststroke aphasia patients. The treatment seems to be beneficial for patients with frontal language area damage, mostly in the distant time after finishing rTMS procedure. |
| **41.** Thiel, Hartmann, Rubi-Fessen, Anglade, Kracht, Weiduschat, Kessler, Rommel, Heiss (2013)  Subacute stroke, Speech (aphasia) | rTMS then therapy  N=24 | Low | 10 day protocol  20 min, 1 Hz rTMS, 90% of rMT | Triangular part of right posterior inferior frontal gyrus (active group)  OR  Midline of vertex (control group) | 45 min. of deficit-specific aphasia therapy delivered by blinded therapist | (+) Ten sessions of inhibitory rTMS over the right posterior inferior frontal gyrus, in combination with speech and language therapy, significantly improve language recovery in subacute ischemic stroke and favor recruitment of left-hemispheric language networks. |
| **42.** Seniow, Waldowski, Lesniak, Iwanski, Czepiel, Czlonkowka (2013)  Subacute stroke, Speech (aphasia) | rTMS then therapy  N=40 | Low | Mon- Friday for 3 weeks (15 sessions)  1 Hz, 90% of rMT, 1800 pulses  (30 minutes)  Or Sham rTMS | Broca’s area homologue (Pars triangularis) | 45 minutes of speech therapy  Impairment-oriented behavioral therapy focused on the individual linguistic problems. Special emphasis was put on the expression and comprehension of spoken language. | (N) Although language functions improved in both experimental and control groups after 3 weeks, only slight group differences in degree of recovery revealed between patients receiving rTMS and control participants. Follow up revealed that seerely aphasic rTMS patients demonstrated significantly greater improvement than patients receiving sham stimulation in repetition.  *Inhibitory stimulus not effective for all stroke patients but may be beneficial for some.* |
| **43.** Rubi-Fessen, Hartmann, Huber, Fimm, Rommel, Thiel, Heiss (2015)  Subacute stroke, Speech (aphasia) | rTMS then therapy  N=30 | Low | 10 sessions (over 2 week period)  1 Hz, 90% of rMT  Or Sham | Right triangular part of inferior frontal gyrus (center of Brodmann area 45) | 45 minutes of speech therapy focused on reactivation of word retrieval as required in tasks such as oral and written picture naming, picture description, and writing from memory. | (+) This study demonstrated that basic linguistic skills as well as functional communication are bolstered by combining rTMS and behavioral language therapy in patients with subacute aphasia. |
| **44.** Cotelli, Fertonani, Miozzo, Rosini, Manenti, Padovani, Ansaldo, Cappa, Miniussi (2011)  Chronic stroke, Speech | rTMS then therapy  N=3 | High | 4 weeks of rTMS (25 min).  5 sessions, 2000 pulses (40 stimuli/train, 50 trains), 90% of MT    20 Hz rTMS delivered in short periods (2 sec duration) separated by longer periods (28 sec) of no stimulation | left dorsolateral prefrontal cortex | Speech therapy  50 min/day (25 min of speech, 25 min of rTMS).  Repetition and reading of the target word to facilitate naming + an articulatory suppression task+ oral picture naming | (+) long lasting effect of combined rTMS and behavioral therapy was observed in aphasic patients |
| **45.** Momosaki, Abo, Kakuda (2014)  Chronic stroke, Oral motor | rTMS then therapy  N=4 | Low/  High | Stimulation 2x daily  3 Hz for 10 s with a 25 second interval, 20 times per session, alternating between left and right hem. (300 pulses/right, 300 pulses/left)- 1200 pulses per day.  130% of rMT for 1^st^ dorsal interosseous muscle of unaffected hem. | Motor area of left and right cerebral hemispheres at site of pharyngeal muscles. | After rTMS  Individualized indirect 20 min swallowing exercise (incl. oral stretching, tongue push up exercise, isokinetic shaker exercise)  6 days of combination therapy | (+) B/L cerebral rTMS and intensive swallowing rehab. Resulted in improvement in swallowing function in all patients |
| **46.** Wang, Hsieh, Tsai, Wang, Lin, Chan (2014)  Chronic stroke, Speech (non-fluent aphasia) | Synchronous  **OR**  rTMS then therapy  N=45 | Low | 10 daily sessions  Online group:  1200 pulses, 90% rMT, 1 Hz, 20 min. while doing picture naming activity  Offline group:  20 minutes, 1 Hz followed by picture naming activity  Sham group: concurrent naming activity | Broca homologous (contralesional pars triangularis) | 60 minute session involving speech training 2x/week  Picture naming activity | (+) rTMS combined with verbal tasks yielded favorable outcomes that were of considerable longevity. The results also indicated that the rTMS protocol and language training can be combined to achieve outcomes superior to those obtained when used separately. |
| **47.** Kakuda, Abo, Momosaki, Morooka, (2011) *Brain Injury*  Chronic stroke, aphasia | rTMS then therapy  N=4 | High then low | 18 sessions over 11 days.  6 Hz priming stimulation (5-sec traing with 25 sec intervals between trains for 10 min, total 600 pulses) followed by 1 Hz rTMS (20 min, total of 1200 pulses)  90% of rMT of first dorsal interosseous muscle of the unaffected upper limb | Inferior frontal gyrus in right frontal lobe | 60 min 1:1 training session focused on expressive modality | (+) Increase in correct answer rate after the intervention. Improvement found in both expressive and recessive language modalities in all patients. This protocol was safe and feasible. |
| **48.** Park, Yoon (2015)  Chronic stroke, Cognitive | Unspecified timing of rTMS  N=20 | High | 20 sessions  100% of rMT, 10 Hz. Applied for 5 seconds with 55 sec. resting period, 1000 times per day | Left prefrontal cortex (affected hem. For all patients) | Group 1: traditional rehab + CACR  Group 2: traditional rehab + rTMS  Computer assisted cognitive rehabilitation performed 20 min a day, 3 days per week, 4 weeks (12 sessions)  All subjects also received traditional physical and occupational therapy concurrently. | (N) CACR is more effective than rTMS in improving cognitive function after stroke |
| **49.** Bentwich, Dobronevsky, Aichenbaum, Shorer, Peretz, Khaigrekht, Gandelman, Marton, Rabey  (2011)  Cognition, Alzheimer’s | rTMS and therapy simultaneous  N=8 | High | 5 sessions per week x 6 weeks = 30  2 sessions/week over 3 months (bi-weekly)= 24  TOTAL OF 54 SESSIONS  Days 1,3,5:  2 brain regions treated per day  20 trains, consisting of 2 seconds of 10 Hz each (20 pulses/train)  Days 2,4:  3^rd^ brain region treated with 25 trains, conwsisting of 2 seconds of 10 Hz (20 pulse/train) totaling 1300 pulses  OR SHAM | Broca, Wernicke, and R-dlPFC brain regions (right and left parietal somatosensory association cortex | Cognitive Training-  Cognitive paradigms: syntax and grammar tasks for Broca region  comprehension of lexical meaning and categorizatiojn tasks for Wernicke  Action, naming, object naming and spatial memory tasks for R-dlPFC | (+) rTNS-COG seems a promising effective and safe modality for AD treatment, possibly as good as cholinesterase inhibitors. |
| **50.** Rabey, Dobronevsky, Aichenbaum, Gonen, Gendelman Marton, Khaigrekht (2013)  Cognition, Alzheimer’s | rTMS and therapy simultaneous  N=15 | High | 5 sessions per week x 6 weeks = 30  2 sessions/week over 3 months (bi-weekly)= 24  TOTAL OF 54 SESSIONS  Days 1,3,5:  2 brain regions treated per day  20 trains, consisting of 2 seconds of 10 Hz each (20 pulses/train)  Days 2,4:  3^rd^ brain region treated with 25 trains, conwsisting of 2 seconds of 10 Hz (20 pulse/train) totaling 1300 pulses  OR SHAM | Broca, Wernicke, and R-dlPFC brain regions (right and left parietal somatosensory association cortex | Cognitive Training-  Cognitive paradigms: syntax and grammar tasks for Broca region  comprehension of lexical meaning and categorization tasks for Wernicke  Action, naming, object naming and spatial memory tasks for R-dlPFC  Spatial attention tasks for R-pSAC and L-pSAC | (+) NeuroAD system offers a novel, safe and effective therapy for improving cognitive function in AD. |

Table S1: This table highlights the diversity of approaches for combined therapy to date. Combined therapy appears to be a safe, well tolerated approach that may lead to favorable outcomes across a variety of diagnoses. Through consistent reporting of stimulation and therapeutic parameters, future studies may determine superior strategies for combined therapy across populations.

*Figure S1*

**Figure S1**: The acronym "tDCS" was used in combination with the same search terms as described in the methods of this review.  The search lead to a total of 1,629 hits across all search terms, compared to 1,136 total hits for rTMS. Investigating combined therapy in the context of tDCS is of considerable interest for future research to optimize this intervention.

*Table S2*

| **rTMS** |
| --- |
| Stimulator and coil characteristic (company, model number) |
| Coil position and orientation |
| Location and process of collecting motor threshold (at rest versus active) |
| Target muscle for determining motor evoked potential (MEP), electrode type |
| Stimulation site |
| Subject positioning and environment (reading, sleeping, etc) |
| Pattern of rTMS in relation to behavioral intervention |
| Frequency and intensity of stimuli (incl. number of pulses, trains, inter-train interval, percentage of motor threshold) |
| Session distribution and total number |
| **Behavioral Intervention** |
| Type of therapy |
| Intervention context (inpatient, outpatient, in-home) |
| Clinician training or certification |
| Frequency of therapy, duration of session |
| Description of quantifiable intervention (including number of repetitions, specific task practice) |
| Use of standardized outcome measures |

**Table S2**: **Suggested Framework for Reporting.** We recommend that the following information be considered and reported for combined rTMS and behavioral interventions. More comprehensive, standardized reporting among investigators may lead to improved study design and understanding in the field.
